# The Impact of Perceptual Load on the Non-Conscious Processing of Fearful Faces

**DOI:** 10.1371/journal.pone.0154914

**Published:** 2016-05-05

**Authors:** Lili Wang, Chunliang Feng, Xiaoqin Mai, Lina Jia, Xiangru Zhu, Wenbo Luo, Yue-jia Luo

**Affiliations:** 1 School of Educational Science, Huaiyin Normal University, Huaian, China; 2 National Key Laboratory of Cognitive Neuroscience and Learning, Beijing Normal University, Beijing, China; 3 Department of Psychology, Renmin University of China, Beijing, China; 4 School of Humanities, Jiangnan University, Wuxi, China; 5 Institute of Psychology and Behavior, Henan University, Kaifeng, China; 6 Research Center of Brain and Cognitive Neuroscience, School of Psychology, Liaoning Normal University, Dalian, China; 7 Institute of Affective and Social Neuroscience, Shenzhen University, Shenzhen, China; Southwest University, CHINA

## Abstract

Emotional stimuli can be processed without consciousness. In the current study, we used event-related potentials (ERPs) to assess whether perceptual load influences non-conscious processing of fearful facial expressions. Perceptual load was manipulated using a letter search task with the target letter presented at the fixation point, while facial expressions were presented peripherally and masked to prevent conscious awareness. The letter string comprised six letters (X or N) that were identical (low load) or different (high load). Participants were instructed to discriminate the letters at fixation or the facial expression (fearful or neutral) in the periphery. Participants were faster and more accurate at detecting letters in the low load condition than in the high load condition. Fearful faces elicited a sustained positivity from 250 ms to 700 ms post-stimulus over fronto-central areas during the face discrimination and low-load letter discrimination conditions, but this effect was completely eliminated during high-load letter discrimination. Our findings imply that non-conscious processing of fearful faces depends on perceptual load, and attentional resources are necessary for non-conscious processing.

## Introduction

Facial expressions are an important component of social communication. For example, fearful expressions are powerful signals of danger in the environment. Being aware of imminent danger is essential to survival of most animals [1]. For this reason, rapid detection of fearful expressions has an obvious adaptive advantage, and fearful expressions may therefore be more likely than other stimuli to capture attention.

Because of the adaptive advantage, it is often assumed that fearful expressions can be processed without attention or consciousness. Neuroimaging studies have demonstrated that the amygdala responds to fearful faces when this information is not accessible to consciousness, suggesting that fearful stimuli can be processed automatically [2–5]. Event-related potential (ERP) measures have also provided evidence for the automatic processing of subliminal fearful faces. Previous studies have found five main ERP components modulated by subliminal fearful faces: anterior N1, N170, vertex positive potential (VPP), N2 and P300. The study by Liddell et al. (2004) was one of the early studies to investigate the ERP responses to subliminal fearful faces and found that subliminal fear increased the amplitude of N2, while supraliminal fear enhanced P3 [6, 7]. The authors considered N2 to be an index of the automatic processing of subliminal fearful stimuli. Kiss and Eimer (2008) reported the similar findings of an N2 enhancement to fearful faces presented subliminally, but not supraliminally [8]. Not all studies have shown a pattern of enhanced N2 to subliminal fearful faces, however. Very recently, one group found the opposite effect, showing increased N2 amplitude to supraliminal and enhanced P3 to subliminal fearful faces [9]. Another study found that, compared to neutral faces, fearful faces reduced N2 amplitude in both subliminal and supraliminal conditions[10]. In addition, subliminal fearful faces can be processed at an early stage, as indicated by an N170/VPP enhancement [8, 11]. Moreover, Eimer et al. (2008) found anterior N1 enhancement in response to masked fearful faces only in trials in which participants successfully detected the fearful faces[12]. In all of these studies, the consistent finding was that the neural response to subliminal fearful faces was different from the response to neutral faces, providing evidence that the subliminal fearful faces can be processed by the brain.

Some researchers have proposed that although fearful stimuli can be processed in the absence of consciousness, such processing may not occur automatically, unconstrained by the availability of attentional resources [13]. Since attentional resources are limited, the extent to which irrelevant distractors are processed depends on the perceptual load of the main task [14]. According to perceptual load theory, perceptual load constitutes a necessary condition for selective attention. Specifically, a high perceptual load for relevant information processing exhausts attentional resources and prevents processing of irrelevant information. However, a low perceptual load does not exhaust attentional resources, thus attention can be spared to process task-irrelevant stimuli. This framework explains why emotion may be processed without attention in tasks with low perceptual load. In support of this theory, several fMRI studies have demonstrated are sponse of the amygdala to unattended fearful expressions in low but not high perceptual load conditions [15–20].

The aim of this study was to test whether the non-conscious processing of fearful faces is constrained by the availability of attentional resources. In most non-conscious processing studies, task-related fearful stimuli are presented at the focal point of attention without any competing stimuli. According to perceptual load theory [14], both task-relevant and task-irrelevant stimuli compete for limited attentional resources, and this competition does not rely on the conscious awareness of task-irrelevant stimuli. Recent studies using binocular rivalry have suggested that subliminal perception can be modulated by the perceptual load [21, 22]. For example, Bahramiet al. (2007) found that the processing of subliminal stimuli in human primary visual cortex (V1) depends on perceptual load. V1 activity to subliminal tool images was reduced in a high load condition compared to a low load condition. However, it remains unclear whether processing of subliminal fearful faces can be influenced by perceptual load.

In the present study, we measured ERPs to determine the effects of perceptual load on the non-conscious processing of fearful faces. Perceptual load was manipulated using a letter search task with letters presented at fixation, and facial expressions (fearful or neutral) were presented peripherally and masked by scrambled faces. Participants were instructed to discriminate the letters at fixation or the facial expression in the periphery. To further examine fear-specific ERP effects in response to subliminal faces, we included a face task, in which participants had to indicate whether masked facial expressions were fearful or neutral. We focused on five main ERP components (anterior N1, N170, VPP, N2 and P3). Our hypothesis was that if the non-conscious processing of fearful faces can be modulated by perceptual load, enhanced responses to fearful faces would be attenuated in the high load condition, which consumes greater attentional resources. In contrast, spill-over of spare attentional capacity in the low load condition should result in larger amplitudes for fearful faces than neutral faces. Alternately, similar ERP effects under low and high load conditions would indicate that non-conscious processing of fearful stimuli may be independent of perceptual load.

## Materials and Methods

### Participants

Twenty-six subjects (13 females, 13 males, right-handed, 18–27 years of age) were recruited from Beijing Normal University in China as paid participants. According to self-report, all participants had normal or corrected-to-normal vision and no history of brain injury. The study was approved by Institutional Review Boards of Beijing Normal University. Written informed consents were obtained from all participants.

### Stimuli

A letter string (3.43°× 0.85° visual angle) was presented at fixation with two identical faces (1.84°× 2.14° each) in the left and right periphery (5.95° eccentricity). Faces were black and white photographs taken from the native Chinese Facial Affective Picture System [23]. A total of 60 grayscale pictures of different individual faces were used. Target face stimuli consisted of 20 fearful faces and 20 neutral faces. Another different 20 neutral faces were used to generate scrambled face masks by dividing each image into a 6×6 matrix of tiles and then randomly rearranging the tiles. Males and females were represented equally among the pictures. The letter strings comprised of six upper-case letters and included target (N or X) and nontarget letters (H, K, M, W, or Z). Low-load letter strings were made of 6 Xs or 6 Ns, whereas high load strings consisted of one of the target letters and five nontarget letters in random order (e.g. ‘NHKWZM’). There was a 50% chance for the target letter to be either an ‘X’ or an ‘N’. Such letter strings have been used in previous studies [17, 24, 25], and are considered to be valid in the manipulation of perceptual load.

### Procedure

**Fig 1:**
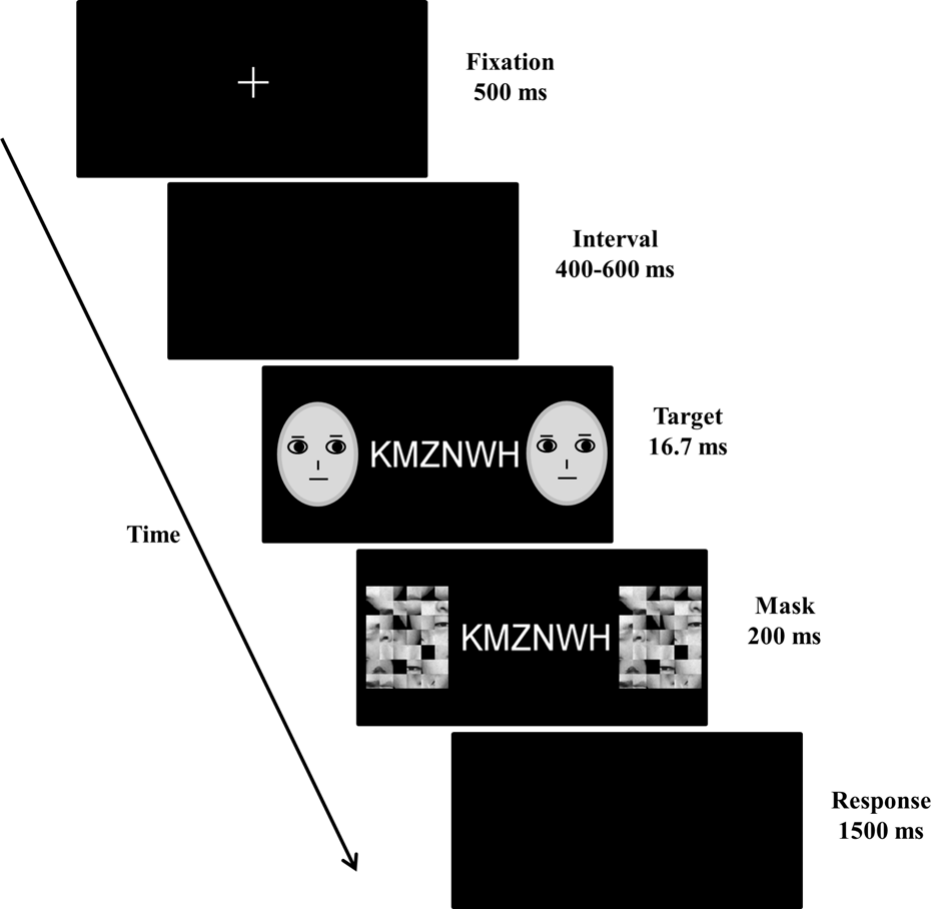
Example of stimulus sequence. On each trial, subjects fixated and viewed a string of 6 letters presented in the centre of the screen, and facial expressions were presented peripherally for 16.7 ms and masked by scrambled faces. The letter string comprised six identical (low load) or six different letters (high load). Participants were instructed to discriminate the letters (X or N) at fixation or the facial expression (fearful or neutral) in the periphery. Please note that schematic faces displayed in the figure were not employed in the experiment but were only used for illustration purpose due to issues of copyrights. Real facial expressions were employed as stimuli in the experiment.

E-Prime software (Psychology Software Tools, Pittsburgh, PA) was used for stimulus presentation and behavioral response collection. The present study consisted of 6 blocks, each containing 100 trials. In each trial (see Fig 1), an initial fixation was presented for 500 ms. After an interstimulus interval (ISI) between 400 and 600 ms, two identical faces were presented together with a letter string for 16.7 ms. The faces were then masked by scrambled faces (200 ms duration), followed by a blank screen which would not disappear until a button-press or until 1500 ms elapsed. The intertrial interval was 500 ms. Participants were required to indicate whether the target letter was an ‘X’ or an ‘N’ in 4 of the blocks (letter task) or whether the masked facial expression was fearful or neutral in the other 2 blocks (face task) by pressing “F” or “J” on the keyboard with their left or right index fingers, respectively. Participants were instructed to respond to the targets as quickly and accurately as possible. At the beginning of each block, an instruction display was presented to instruct participants as to whether they would be performing the face task or the letter task. Therefore, the focus of their attention (on letters or faces) changed between blocks. The sequence of six blocks was randomized for each participant. In each block, high- and low-load letter strings appeared randomly and with equal probability, and half of the faces were fearful, the other half were neutral.

Participants were seated in a dimly lit and sound-attenuated room, with their eyes approximately 100 cm away from the screen. Stimuli were presented on a 21-inch CRT monitor (60 Hz refresh rate).To ensure the participants maintained vigilance for the face task, it was emphasized that although the masked face would be difficult to see, they had to attend to faces in preparation for post-testing briefings. We actually did not have briefings after the test. This method was often used in the subliminal studies [6, 7].

### EEG recording

Electroencephalogram (EEG) was recorded from 64 scalp sites using Ag/AgCl electrodes mounted in an elastic cap (NeuroScan Inc., Herndon, Virginia, USA), with the reference on the left mastoid. Vertical electrooculogram (VEOG) was recorded from electrodes above and below the left eye, and horizontal electrooculogram (HEOG) was recorded from electrodes placed at the outer canthi of both eyes. All interelectrode impedance was maintained below 5kΩ. The EEG and EOG were amplified using a 0.05–100 Hz bandpass filter and continuously sampled at 500 Hz/channel. The EEG data was low-pass filtered offline below 30 Hz and re-referenced to the global average reference. Trials containing blinks, eye movements, or other artifacts (EEG sweeps with amplitudes exceeding ±80μV) were excluded.

### Data analysis

The ERPs for the target stimuli were analyzed in the present study. The ERP waveforms were time-locked to the onset of the target stimuli. Separate EEG epochs of 1000 ms (200 ms baseline) were extracted offline for the stimuli. In the letter task, only trials with correct behavioral responses were included in the average. In the face task, because the discrimination performance of the target faces was at chance level, ERPs were collapsed across trials in which participants responded correctly or incorrectly [8]. The mean number of trials contributing to ERP averages for fearful faces in face, low-load, and high-load conditions was 98, 94, and 71, respectively, and for neutral faces was also 98, 94, and 71, respectively.

Based on the results of previous ERP studies [6–8, 11], anterior N1, N170, VPP, N2, and P3 components were measured. Through visual inspection of the grand-average and previous studies [8, 9], the baseline-to-peak amplitudes of anterior N1 (80–140 ms) and VPP (140–190 ms) and the mean amplitudes of N2 (250–330 ms) were computed at the following 9 electrode sites: F3, Fz, F4, FC3, FCz, FC4, C3, Cz, C4; the mean amplitude of P3 (450–700 ms) was computed at FC3, FCz, FC4, C3, Cz, C4, CP3, CPz, CP4, P3, Pz, and P4. Four-way repeated measures analysis of variance (ANOVA) were conducted for these components with Task (face *vs*. low load *vs*. high load), Expression (fearful *vs*. neutral), Hemisphere (left *vs*. midline *vs*. right), and Electrode as within-subject factors. In addition, the baseline-to-peak amplitude of N170 component was measured at occipito-temporal sites (P7, P8, PO7, and PO8) as the maximal negative peak in the time window of 150–190 ms. The N170 amplitude was then analyzed using a 3 × 2 × 2 × 2 four-way repeated measures analysis of variance with Task (face *vs*. low load *vs*. high load), Expression (fearful *vs*. neutral), Hemisphere (left *vs*. right), and Electrode (parietal: P7 and P8 *vs*. parieto-occipital: PO7 and PO8) as within-subject factors. Greenhouse-Geisser corrected degrees of freedom were used whenever appropriate[26]. Post-hoc testing of significant main effects was conducted using Bonferroni method.

## Results

### Behavioral results

In the letter detection task, mean reaction times (RTs) and accuracy data were submitted to a 2 × 2 two-way repeated measures ANOVA with factors of Load (low load *vs*. high load) and Expression (fearful *vs*. neutral). Analysis of RTs revealed a significant main effect of Load, *F* (1, 25) = 295.03, *p* < 0.001, *η*_*p*_^*2*^ = 922, participants responded faster in the low-load task (M ± SE, 308.18 ± 15.46 ms) than in the high-load task (620.03 ± 25.24 ms). The results revealed no effect of Expression (*F* (1, 25) = 0.15, *p* > 0.69), and no interaction on RTs (*p*> 0.87). Analysis of accuracy revealed a significant main effect of Load, *F* (1, 25) = 730.23, *p* < 0.001, *η*_*p*_^*2*^ = 0.967. The accuracy was higher in the low-load task (96.10 ± 0.68%) compared to the high-load task (72.65 ± 1.06%). We did not find the significant main effect of Expression (*F* (1, 25) = 0.001, *p* > 0.97), or interaction (*p* > 0.62).

**Fig 2:**
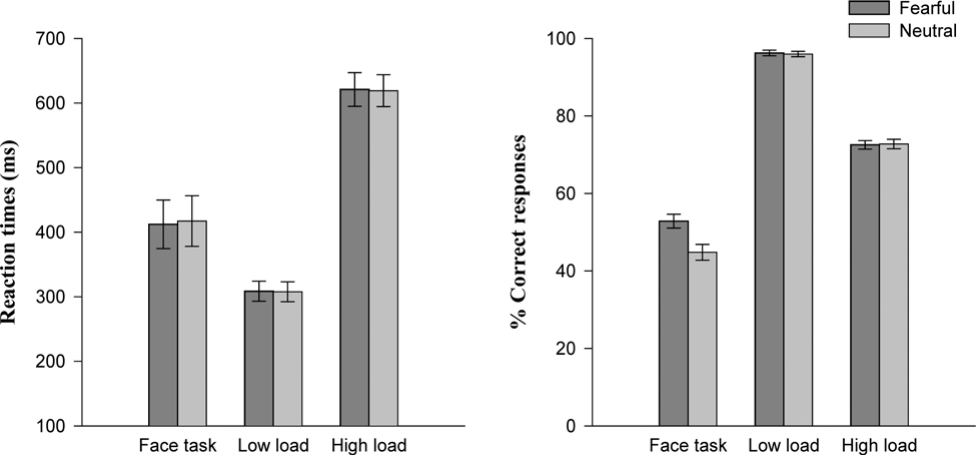
Reaction times and percentages of correct responses in the face task and letter task across low or high perceptual load and fearful or neutral peripheral facial expressions. Error bars show one standard error (S.E).

In the face task, mean RTs for trials with correctly reported fearful faces and neutral faces were 412.22 ± 37.64 ms and 417.30 ± 39.19 ms, and the difference between facial expressions was not significant, *t* (25) = -0.76, *p* > 0.45. The accuracy was higher for fearful faces (52.85 ± 9.10%) than neutral faces (44.81 ± 10.49%), *t* (25) = 2.43, *p* = 0.023. See Fig 2 for behavioral results.

To obtain an objective estimate of participants’ ability to detect masked fearful faces in the face task, *d′* values (Macmillan and Creelman, 1991) were measured on the basis of hits (correct responses on trials with fearful targets) and false alarms (incorrect responses on trials with neutral targets). The mean *d′* (-0.05) did not significantly differ from zero (t (25) = -1.25, *p* > 0.22), suggesting that participants’ performance of face discrimination was at the level of chance. These data suggest that participants were not able to categorize the subliminal facial expressions above chance. Response bias *c* (Macmillan and Creelman, 1991) was also computed and showed that participants were more likely to report fearful faces (*c* = -0.11; t (25) = -2.45, *p* = 0.022). The result of accuracy analysis showed a high accuracy for fearful faces (fearful: 52.85%; neutral: 44.81%), which may be due to participants having a higher tendency to answer "fear" when in doubt.

### ERP results

#### Anterior N1

**Fig 3:**
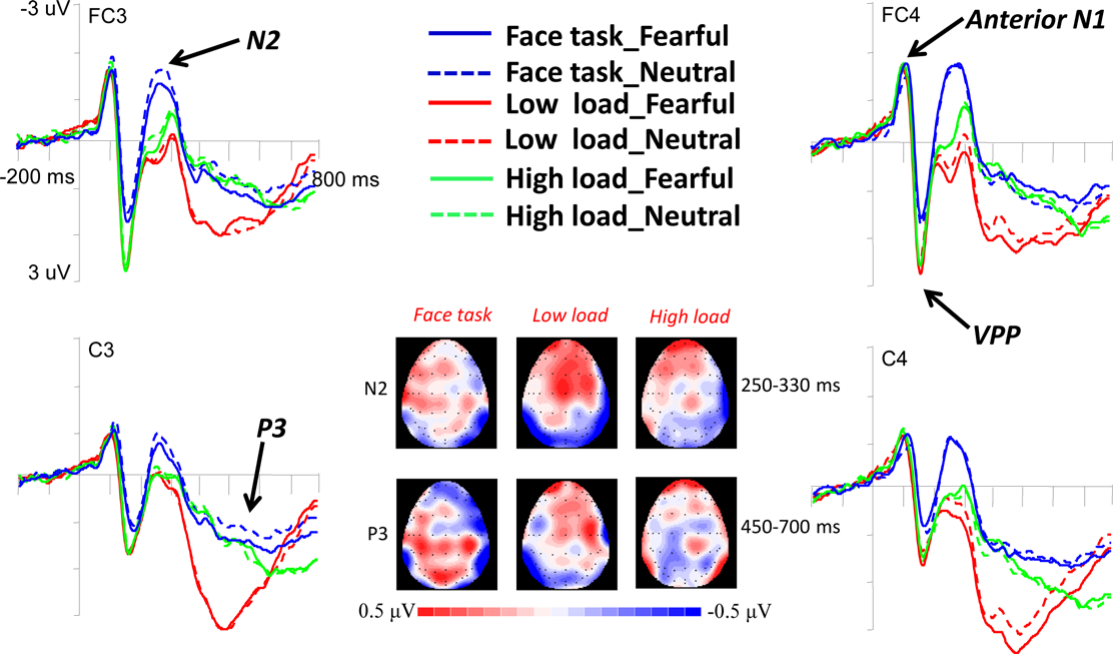
Grand average ERP waveforms elicited by masked fearful faces and masked neutral faces in the face task and letter task across low or high perceptual load at fronto-central and central electrodes. The topographic maps at the bottom display differences between the ERPs for fearful and neutral stimuli in the time window of 250-330ms and 450–700 ms.

Anterior N1 is an early anterior negativity (80-140ms) and the anterior part of the visual P1. Four-way repeated measures ANOVA of Task (face *vs*. low load *vs*. high load) by Expression (fearful *vs*. neutral) by Hemisphere (left *vs*. midline *vs*. right) by Electrode (frontal: F3, Fz, and F4 *vs*.fronto-central: FC3, FCz, and FC4 *vs*. central: C3, Cz, and C4) on anterior N1 amplitudes yielded a significant main effect at Electrode, *F* (1.17, 29.17) = 8.03, *p* = 0.006, *η*_*p*_^*2*^ = 0.243. Post-hoc tests showed that the anterior N1 amplitude was significantly more negative for fronto-central sites (-2.44 ± 0.22 μV) relative to central sites (-1.95 ± 0.18 μV; *p* < 0.001), but there was no differences between frontal (-2.36 ± 0.26 μV) and fronto-central sites, or between frontal and central sites (all *p*s > 0.05). The main effect of Hemisphere also reached significance (*F* (2, 50) = 40.91, *p* < 0.001, *η*_*p*_^*2*^ = 0.621), and post hoc tests showed that the amplitude was enhanced for midline (-2.87 ± 0.26 μV) compared to the left (-1.89 ± 0.19 μV) and right (-1.99 ± 0.20μV) hemispheres (all *p*s < 0.05). The Electrode × Hemisphere interaction reached significance, *F* (2.40, 59.94) = 17.01, *p* < 0.001, *η*_*p*_^*2*^ = 0.405, showing that frontal and fronto-central sites elicited larger amplitudes than central sites over both the left and right hemisphere (all *p*s < 0.05), but no difference between frontal and fronto-central sites was observed (all *p*s > 0.05); over the midline, the amplitudes were higher for FCz than Fz and Cz (all *p*s < 0.05) with no difference between Fz and Cz (*p* > 0.05). There was no significant effect involving task and expression (see Fig 3).

#### N170

**Fig 4:**
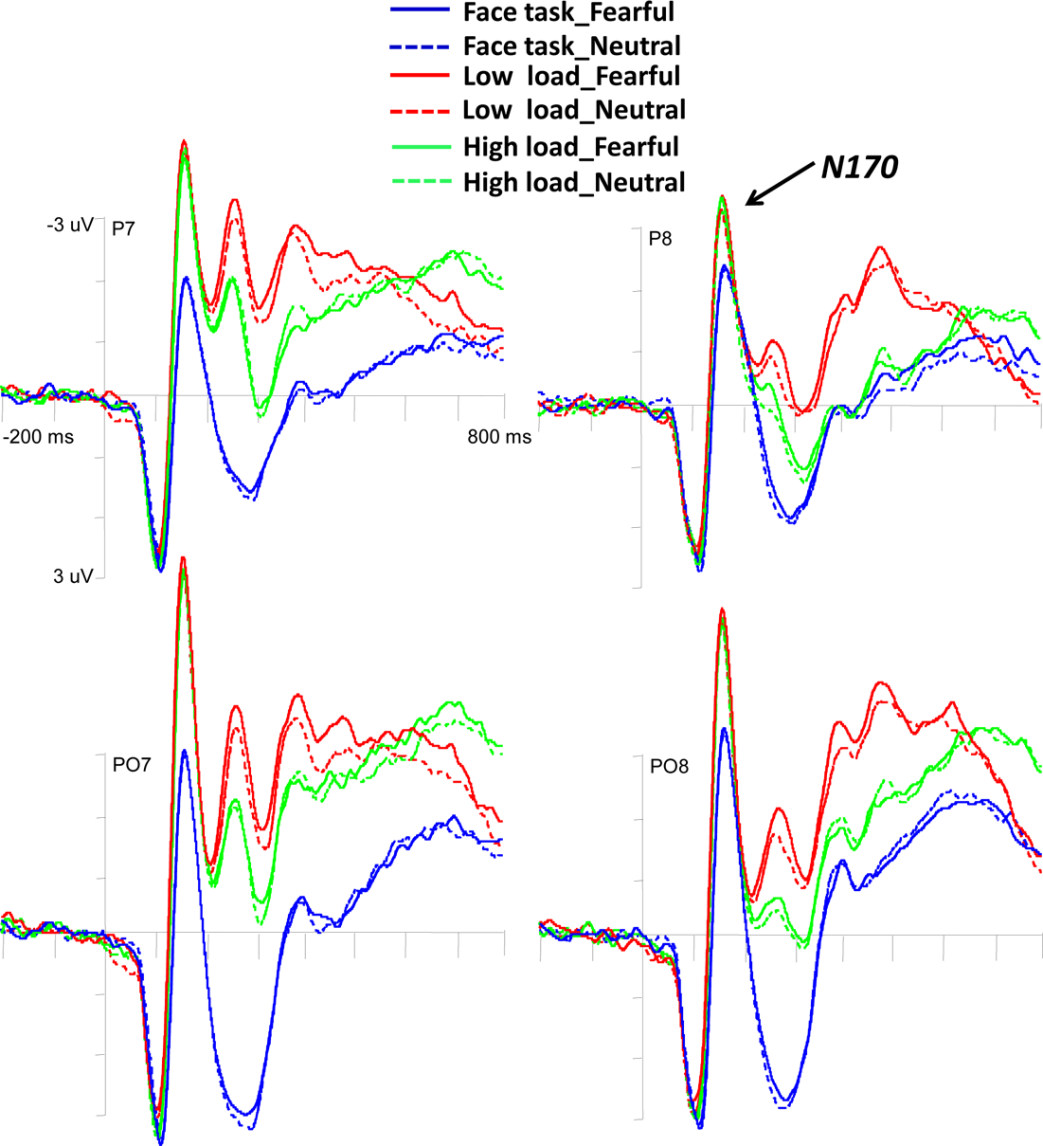
Grand average ERP waveforms elicited by masked fearful faces and masked neutral faces in the face task and letter task across low or high perceptual load at lateral occipito-temporal electrodes.

Four-way repeated measures ANOVA of Task (face *vs*. low load *vs*. high load) by Expression (fearful *vs*. neutral) by Hemisphere (left *vs*. right) by Electrode (parietal: P7 and P8 *vs*.parieto-occipital: PO7 and PO8) on N170 amplitudes yielded a main effect of Task (*F* (1.20, 29.91) = 60.16, *p* < 0.001, *η*_*p*_^*2*^ = 0.706), indicating that both low load (-5.23 ± 0.54 μV) and high load (-5.14 ± 0.53 μV) elicited larger negativity than the face task (-3.18 ± 0.52 μV). The main effect of Electrode also reached significance, *F* (1, 25) = 24.13, *p* < 0.001, *η*_*p*_^*2*^ = 0.491, showing that the amplitudes were more negative for parieto-occipital electrodes (-5.37 ± 0.66 μV) than parietal electrodes (-3.67 ± 0.40 μV). Moreover, the interaction of Expression by Hemisphere by Electrode was significant, *F* (1, 25) = 7.84, *p* = 0.01, *η*_*p*_^*2*^ = 0.239, indicating that the N170 amplitude was marginally larger for fearful faces (-3.53 ± 0.37 μV) compared to neutral faces (-3.35 ± 0.39 μV) at electrode P8 (*p* = 0.07), but no difference between fearful faces and neutral faces was found at electrode P7, PO7 and PO8 (all *p*s > 0.11) (see Fig 4).

#### VPP

Four-way repeated measures ANOVA of Task (face *vs*. low load *vs*. high load) by Expression (fearful *vs*. neutral) by Hemisphere (left *vs*. midline *vs*. right) by Electrode (frontal: F3, Fz, and F4 *vs*.fronto-central: FC3, FCz, and FC4 *vs*. central: C3, Cz, and C4) on VPP amplitudes yielded a significant effect of Task (*F* (1.30, 32.45) = 38.59, *p* < 0.001, *η*_*p*_^*2*^ = 0.607), indicating that the amplitudes were smaller for the face task (2.03 ± 0.33 μV) than low-load (2.97 ± 0.33 μV) and high-load (3.02 ± 0.35 μV) tasks (all *p*s < 0.001), and no difference between low and high load tasks was observed (*p* > 0.47). The main effect of Electrode also reached significance (*F* (1.14, 28.58) = 27.88, *p* < 0.001, *η*_*p*_^*2*^ = 0.527), and post hoc tests demonstrated larger amplitudes at frontal sites (3.32 ± 0.39 μV) than fronto-central (2.77 ± 0.36 μV) and central (1.92 ± 0.29 μV) sites, and the difference between the latter two sites also reached significance (all *p*s < 0.01) (see Fig 3).

#### N2 (250-330ms)

Four-way repeated measures ANOVA of Task (face *vs*. low load *vs*. high load) by Expression (fearful *vs*. neutral) by Hemisphere (left *vs*. midline *vs*. right) by Electrode (frontal: F3, Fz, and F4 *vs*.fronto-central: FC3, FCz, and FC4 *vs*. central: C3, Cz, and C4) on N2 amplitudes yielded main effects of Task, Expression, and Hemisphere (*F* (2, 50) = 35.20, *p* < 0.001, *η*_*p*_^*2*^ = 0.585; *F* (1, 25) = 7.74, *p* = 0.01, *η*_*p*_^*2*^ = 0.236; *F* (2, 50) = 15.39, *p* < 0.001, *η*_*p*_^*2*^ = 0.381). The face task (-1.44 ± 0.29 μV) elicited larger negativity than low load (0.20 ± 0.31 μV) and high load (-0.63 ± 0.30 μV), and the difference between the latter two tasks also reached significance (all *p*s < 0.01). In addition, the amplitudes were more negative for neutral faces (-0.69 ± 0.27 μV) compared with fearful faces (-0.55 ± 0.29 μV). Increased amplitudes were observed over midline (-1.25 ± 0.36 μV) compared to left (-0.29 ± 0.25 μV) and right (-0.33 ± 0.28 μV) hemisphere (all *p*s < 0.001), and no difference was found between the left and right hemisphere (*p* > 0.99).Importantly, there was also a Task × Expression × Hemisphere interaction (*F* (4, 100) = 2.68, *p* = 0.036, *η*_*p*_^*2*^ = 0.097). Further analysis revealed that the N2 amplitudes were more positive for fearful than neutral faces in the face and low-load tasks (all *p*s < 0.05), but no difference in N2was observed between facial expressions in the high-load task (all *p*s > 0.05). In the face task, the N2 difference between facial expressions was observed over the left hemisphere (fearful, -0.97 ± 0.28 μV; neutral, -1.13 ± 0.29 μV; *p* = 0.039), but not observed over the midline (fearful, -1.92 ± 0.37 μV; neutral, -2.02 ± 0.38 μV; *p* > 0.38) or right hemisphere (fearful, -1.31 ± 0.29 μV; neutral, -1.29 ± 0.24 μV; *p* > 0.73). In the low load condition, the N2 difference between facial expressions was observed over both the midline (fearful, -0.05 ± 0.41 μV; neutral, -0.42 ± 0.37 μV; *p* = 0.004) and right hemisphere (fearful, 0.68 ± 0.33 μV; neutral, 0.36 ± 0.34 μV; *p* = 0.013), but not over the left hemisphere (fearful, 0.36 ± 0.29 μV; neutral, 0.28 ± 0.29 μV; *p* > 0.45) (see Fig 3).

#### P3 (450-700ms)

Four-way repeated measures ANOVA of Task (face *vs*. low load *vs*. high load) by Expression (fearful *vs*. neutral) by Hemisphere (left *vs*. midline *vs*. right) by Electrode (fronto-central: FC3, FCz, and FC4 *vs*. central: C3, Cz, and C4 *vs*. centro-parietal: CP3, CPz, and CP4 *vs*. parietal: P3, Pz, and P4) on P3 amplitudes revealed a main effect of Task, *F* (2, 50) = 23.72, *p* < 0.001, *η*_*p*_^*2*^ = 0.487. As shown in Fig 3, Post-hoc tests indicated that P3 amplitude was greater in the low load task (1.84 ± 0.13 μV) than in face (0.80 ± 0.14 μV) and high load tasks (1.22 ± 0.17 μV; all *p*s < 0.01), while the latter two tasks did not show significant differences (*p* > 0.068). Also the main effect of Electrode was observed (*F* (1.23, 30.68) = 10.58, *p* = 0.002, *η*_*p*_^*2*^ = 0.297). Post-hoc tests indicated that P3 amplitude was greater at the central sites (2.00 ± 0.19 μV) than fronto-central sites (1.35 ± 0.24 μV; *p* = 0.001) and parietal sites (0.33 ± 0.27 μV; *p* = 0.001), while there was no difference between central sites and centro-parietal (1.47 ± 0.18 μV, *p* > 0.15). Moreover, a significant Task × Expression × Hemisphere interaction was observed, *F* (4, 100) = 2.64, *p* = 0.038, *η*_*p*_^*2*^ = 0.095. Post-hoc tests showed that fearful faces elicited larger P3 amplitudes than neutral faces in the face and low-load tasks, but this effect was only observed over the left hemisphere in the face task (fearful, 0.86 ± 0.15 μV; neutral, 0.60 ± 0.13 μV; *p* = 0.013) and over the right hemisphere in the low-load task (fearful, 1. 72 ± 0.17 μV; neutral, 1.51 ± 0.17 μV; *p* = 0.016). The effect was completely eliminated in the high-load task (all *p*s > 0.26).

## Discussion

To examine whether processing of subliminal fearful faces is constrained by attentional resources, we manipulated perceptual load by having participants perform simple and difficult letter search tasks. Two identical faces (either neutral or fearful) were presented peripherally and masked by scrambled faces. Participants were unable to categorize the faces by emotional expression. ERP results in the face task showed that fearful faces were associated with reduced N2 amplitude and increased P3 amplitude relative to neutral faces. The perceptual-load manipulation was highly effective in both behavioral and ERP responses, as participants were consistently faster and more accurate at detecting letters in the low-load task than in the high-load task. Similar to the face task, the low-load task was associated with reduced N2 and increased P3 amplitudes in response to fearful faces relative to neutral faces. However, no changes in ERP components were observed during high perceptual load. Our results suggest that processing of subliminal fearful faces depends on perceptual load.

Based on ERP findings in the face task and low-load task, N2 and P3 appear to be sensitive to the influence of perceptual load upon non-conscious processing of fearful faces. Previous studies have found these two components to be modulated by subliminal or supraliminal conditions with a great deal of variability across studies. Most reports have shown that enhanced N2 to fearful faces in subliminal but not supraliminal conditions, and the N2 effect was therefore interpreted as an index of a non-conscious attention-orienting response to fearful faces [6–8]. Some researchers also reported that a P3 enhancement to fearful faces was found in supraliminal but not subliminal conditions, suggesting that P3 might be related to conscious processing of emotional stimuli [6, 8, 11]. However, other studies observed no difference in N2 between subliminal fearful and neutral faces [9, 11], or enhanced P3 to fearful faces on subliminal trials [9]. In contrast to previous investigations, our study showed an enhanced positivity for fearful faces relative to neutral faces from about 250 ms to 700 ms after stimulus onset. That is, reduced N2 and enhanced P3 to fearful faces were revealed in the face task and low-load task. In line with our findings, a recent study by Smith (2012) revealed that N2 amplitude was reduced for fearful faces relative to neutral faces, regardless of whether the faces were subliminal or supraliminal[10]. Enhanced positivity for fearful faces has often been observed under supraliminal conditions [27–31]. Such modulation may reflect the elaboration and context evaluation of the incoming emotional signals [31, 32]. Our findings suggest that such emotional positivity may not be consciousness-specific, but can also be elicited by non-conscious stimuli. Balconi and Mazza (2009) also reported that P3 amplitudes were increased for subliminal facial expressions with high arousal (e.g., fear, happiness, surprise) compared to faces with low arousal (e.g., neutral, sadness) [9]. These ERP effects imply that the brain can detect subliminal threat information, which is adaptive for human survival.

An unexpected finding was the distinct topographic differences between fearful and neutral faces in different tasks. For the face task, the emotional positivity effects of fearful faces (N2, P3) were greater in the left hemisphere; for the low-load task, these effects were greater in the right hemisphere. For decades, many researchers have investigated brain lateralization of emotional processing, and the dominance of the right hemisphere has been emphasized in negative facial expression processing, while some studies have also supported left brain activity [33, 34]. The specific pattern of brain activation observed during face and low-load tasks in the current study may have been due to the task demands, which determined whether facial expressions were processed in the focus of attention or the periphery. In the face task, participants were required to discriminate facial expressions in the periphery, and the faces were processed with sufficient attentional resources. In the low-load task, participants performed a letter discrimination task at the focal point, and fewer attentional resources remained for processing peripheral faces. In other words, task demands influenced the degree of attentional resources allocated to process faces, and might have affected brain activation. Research by Hardee et al. (2008) has suggested that the left and right amygdala play different roles in emotional processing [35]. The right amygdala may act as a course detector of overall change, focusing automatically and non-selectively on potential threat signals [3], while the left amygdala is sensitive to detailed information of emotional expression (e.g., gaze direction, eye white area) and differentiates between emotional expressions, demonstrating a higher level of discrimination [35]. The emotional positivity for fearful faces in our study might reflect a contribution of subcortical (e.g. amygdala) modulation of cortical activity [36]. In the face task, plenty of available attentional resources made the details of emotional expressions (e.g., eyes, mouth) available for processing and the left amygdala may have been more involved in processing than the right amygdala. In contrast, the right amygdala might be activated in low load conditions with less attentional resources to process fearful faces, and this process might be coarse and automatic. Our ERP effect in low load condition was consistent with a previous fMRI study [20], which found that unattended fearful faces activated the right but not left amygdala in the low-load task. Furthermore, the left frontal regions (e.g., anterior cingulated, inferior frontal, medial frontal), used for alerting one to significant and novel stimuli, can be activated in response to non-conscious emotional faces presented inside the attentional focus [37, 38]. However, the anatomical level of the specific ERP effects cannot be inferred from the present study. Further source analyses and fMRI studies are needed to determine the role of the left and right hemisphere in emotional processing.

A fundamental question in the study of emotion is its relationship to attention and awareness. Previous reports have shown that processing of emotional stimuli could be affected by perceptual load when stimuli were presented under supraliminal conditions [15–17, 19, 20]. In the present study, we focused on the relationship between non-conscious processing of fear and perceptual load. From an evolutionary perspective, detecting fearful faces might provide important social cues that enhanced the survival of the individual, thus increasing the chances of survival of the species. Therefore, fearful faces may have a special ability to reduce the effect of perceptual load on non-conscious processing. However, all of these ERP effects found within the face and low-load tasks were completely eliminated in the high-load task. ERP modulation by perceptual load may suggest that attentional capacity constitutes a necessary condition for the perception of emotional information, even when this information is not consciously perceived. The automatic processing of emotional stimuli depends on the extent to which the main task depletes attentional resources. ERP effects in the low-load task may be triggered by spare attentional capacity, that allows masked fearful faces to be processed. Our results suggest that the effects of perceptual load are not restricted to the neural representation of emotional stimuli that have reached conscious awareness and extend load theory to non-conscious processing.

In addition, our study did not find an early ERP effect (anterior N1, N170, VPP) related to the subliminal emotional processing. Recent studies have indicated that the electrophysiological differences between fearful and neutral faces occur early (within 200 ms). For example, subliminal fearful faces elicited larger N1 amplitudes than neutral faces, but only on correct trials [12], and enhanced VPP amplitudes were also observed for subliminal fearful faces [8]. Modulation of N170 by fearful faces was also observed in the subliminal condition, meaning that fearful faces elicited larger amplitudes than neutral faces [11], even when the faces were presented outside the attentional focus [39]. However, some researchers found that modulation of N170 by emotional valence was only observed when participants could reliably detect the fearful stimulus [10, 40]. Compared to previous subliminal studies, the task demands and the location of the faces in the present study were distinct. Esslen et al. (2004) proposed that minor difference in experimental design might cause different results [36]. Thus, the reason for this discrepancy between the current study and previous studies needs to be explored in future experiments.

## Conclusions

The present study investigated whether non-conscious processing of emotional stimuli depends on perceptual load. An emotional positivity was observed over the frontal and central areas from about 250 ms to 700 ms after stimulus onset in the face task and low-load tasks, but not in the high-load task. Our findings suggest that attentional resources are needed for the non-conscious processing of fearful faces. This provides initial evidence that perceptual load may affect the mechanisms underlying non-conscious processing of emotional stimuli.
